# Corrosion and Corrosion Fatigue Properties of Additively Manufactured Magnesium Alloy WE43 in Comparison to Titanium Alloy Ti-6Al-4V in Physiological Environment

**DOI:** 10.3390/ma12182892

**Published:** 2019-09-07

**Authors:** Nils Wegner, Daniel Kotzem, Yvonne Wessarges, Nicole Emminghaus, Christian Hoff, Jochen Tenkamp, Jörg Hermsdorf, Ludger Overmeyer, Frank Walther

**Affiliations:** 1 Department of Materials Test Engineering (WPT), TU Dortmund University, Baroper Str. 303, D-44227 Dortmund, Germany; 2 Materials and Process Department, Laser Zentrum Hannover e.V. (LZH), Hollerithallee 8, D-30419 Hannover, Germany

**Keywords:** additive manufacturing, laser powder bed fusion (L-PBF), magnesium alloy WE43, titanium alloy Ti-6Al-4V, biomaterials, corrosion, in vitro fatigue

## Abstract

Laser powder bed fusion (L-PBF) of metals enables the manufacturing of highly complex geometries which opens new application fields in the medical sector, especially with regard to personalized implants. In comparison to conventional manufacturing techniques, L-PBF causes different microstructures, and thus, new challenges arise. The main objective of this work is to investigate the influence of different manufacturing parameters of the L-PBF process on the microstructure, process-induced porosity, as well as corrosion fatigue properties of the magnesium alloy WE43 and as a reference on the titanium alloy Ti-6Al-4V. In particular, the investigated magnesium alloy WE43 showed a strong process parameter dependence in terms of porosity (size and distribution), microstructure, corrosion rates, and corrosion fatigue properties. Cyclic tests with increased test duration caused an especially high decrease in fatigue strength for magnesium alloy WE43. It can be demonstrated that, due to high process-induced surface roughness, which supports locally intensified corrosion, multiple crack initiation sites are present, which is one of the main reasons for the drastic decrease in fatigue strength.

## 1. Introduction

Due to higher life expectancy, people are facing new diseases, which can limit the quality of life. Therefore, a great deal of research activity has been carried out in the last century and the overall aim of these purposes has been superior medical solutions like biomedical implants and improving the perspectives of the patient [1]. Concerning the potential implant, only a few material classes can fulfill the challenging requirements. However, several metallic materials have been used since the early 1900s as scaffolds and for load-bearing implants [2]. Typical biomedical applications are wires, screws for fracture fixation plates, and joint prostheses for hips, knees, or shoulders [2]. Further on, metallic implants were used in cardiovascular surgery and dental medicine. The most commonly favored metals in the medical sector are stainless steel, titanium and its alloys, and cobalt-based as well as tantalum-based alloys [2].

In general, titanium alloys are preferred due to their high strength-to-weight ratio and corrosion resistance [3,4]. Further on, they show good biocompatibility due to the thin oxide layer which forms rapidly on the surface [5]. Titanium alloys show in vivo and in vitro superior corrosion resistance under static loading conditions due to the presence of the passive film on the surface [6]. Even if the passive film is damaged, repassivation takes place and limits metal dissolution. However, it has been demonstrated that cyclic loading negatively influences the metal dissolution as well as the repassivation kinetics [6]. As a consequence, the continuous breakdown of the passive film causes localized corrosion, which leads to decreased fatigue life and, further on, toxic metallic ions are released [7]. Another important drawback of titanium alloys as a biomaterial is the stiffness mismatch between the implant and the human bone, resulting in stress shielding which can cause bone resorption, resulting in loosening the implant. Thereby, different approaches, like the development of complex lattice structures or new materials, have been pursued in recent years. In this context, magnesium alloys are promising candidates with applications in the field of stents as well as reconstruction and fracture plates [8]. This is due to the natural occurrence of magnesium in the human body, the participation in many processes [9], and the similar mechanical properties to the human cortical bone preventing stress shielding [10]. Furthermore, the body can excrete it without harmful side effects, even at high concentrations [9]. A challenge with pure magnesium, however, is the excessively high and unpredictable corrosion rate under physiological environmental conditions with an associated release of hydrogen gas [11,12]. Also, the rapid degradation leads to a reduction of the cross-section and to the formation of corrosion scars, which drastically reduces the stability of the implant. With simultaneous mechanical loading, crack initiation occurs at these sites, which in turn are also a favored site for a corrosive attack so that these two effects reinforce each other [13,14,15].

The in vitro determination of corrosion rates is not a trivial issue. The corrosion behavior is influenced by the alloy composition and the microstructure [16]. Conventional methods such as potentiodynamic polarization (PDP) measurement provide inaccurate characteristic values by determining the corrosion current density and significantly underestimating the corrosion rate. The reason for this is the so-called negative difference effect (NDE), which, according to recent studies, can be explained by a cathodic activation of the samples under anodic polarization, so that surface films are formed which have a persistent cathodic effect. Consequently, the net anodic current density is reduced by a cathodic value [17,18,19]. Consequently, the classical immersion test with a detection of the hydrogen volume formed and a measurement of the weight reduction is the most accurate way to determine the corrosion rate.

There are few studies in the field of fatigue behavior of magnesium as a biomaterial. Many of them refer to the low cycle fatigue range [20,21,22,23], and only a few studies with superposition through body-like conditions could be found [23]. Above all, there is a lack of studies on the in vitro fatigue behavior of magnesium alloys containing rare earth elements (RE). One study series deals with the corrosion fatigue behavior of the magnesium alloy ZX10 with different extrusion temperatures in a simulated body fluid (SBF) [24], and a second publication deals with the corrosion fatigue behavior of different alloys under static and dynamic environmental conditions [25]. Despite the increasing interest in magnesium as a biomaterial, there is a lack of corrosion fatigue investigations under body-like conditions. 

Additionally, structural circumstances inside the human body are challenging factors for potential implants, not only for the material but also for the manufacturing process. It is favored that implants are customized to the needs of the patient. Due to this, the implant shape has to be highly complex and conventional subtractive manufacturing methods are limited, thus, new manufacturing techniques have to be developed to take into account patients’ requirements. Additive manufacturing (AM) has developed over the past years as a common technique to produce implants or medical devices with complex geometries [26,27]. Due to the high geometrical freedom, personalized implants with local adjusted material properties can be manufactured [28]. Concerning possible AM techniques, which can process titanium and magnesium alloys, especially powder bed fusion (PBF) techniques, using a high power heat source of either laser or electron beams for local melting of powder particles, are favored. Both techniques are based on the layer-by-layer manufacturing of components. In particular, the base plate is covered by a powder layer, which is subsequently melted by the heat source according to the corresponding CAD data. After this, the base plate is lowered and again covered with the powder [29]. This principle enables the manufacturing of highly complex geometries. 

In comparison to electron powder bed fusion (E-PBF), laser powder bed fusion (L-PBF) achieves higher accuracy [28] and better as-built surface roughness [30] but has an increased process time compared to conventional methods [31]. Until now, high surface roughness and process-induced defects, such as porosity or near-surface notch-like defects, are limiting factors. Regarding in vivo applications, near-surface defects can facilitate corrosion processes which can end up in early failure of the implant and increased risk of toxicity [2]. Therefore, the material, especially the damage behavior of the used materials, has to be understood in detail. Much research was carried out in the origin of defects and they can mainly be reduced to process parameters during the manufacturing process [32]. For both types of implants, i.e. permanent and resorbable, representative material classes were manufactured by L-PBF. Despite the different material classes and the resulting areas of application, it should be shown to what extent the mechanical stability of magnesium is inferior to titanium. In the case of the permanent implant class, the Ti-6Al-4V alloy was selected, and for the resorbable implant class, the magnesium alloy WE43 was chosen. The main objective of this work was to highlight the influence of different process parameters on resulting microstructure, process-induced porosity, and corrosion fatigue strength for additively manufactured materials, especially for magnesium alloy WE43, to ensure a reliable application in future medical devices.

## 2. Experimental Methods

### 2.1. Materials

#### 2.1.1. Magnesium

**Figure 1:**
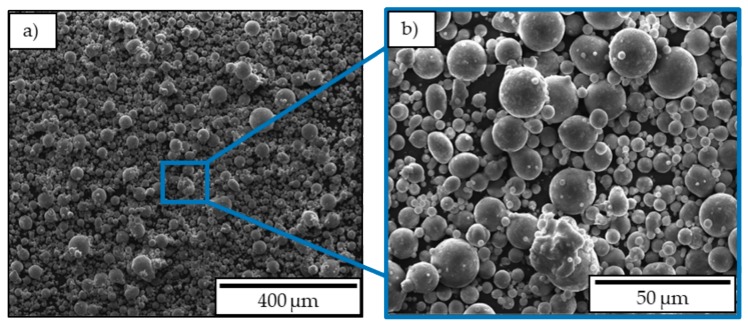
SEM images of a WE43 magnesium powder: (**a**) ×500, (**b**) ×1000 magnification.

Investigations were conducted for two different biomedical materials. A resorbable material (magnesium alloy) was compared to a non-resorbable material (titanium alloy). Concerning magnesium, the powder material WE43 alloyed with yttrium and rare earth elements produced by LPW Technology Ltd (Cheshire, Great Britain) was used. The particle size range is between 20 to 63 µm. SEM (scanning electron microscopy, Thermo Fisher Scientific, Waltham, MA, USA) pictures, shown in Figure 1, reveal spherical particles with particle attachments and high amounts of fines. The biological compatibility of WE43 was confirmed several times, including cell survival rates of 96% (cell line L929), good in vivo integration with surrounding bone (rabbits, 2 months), and no adverse effects due to gas bubbles [33].

**Figure 2:**
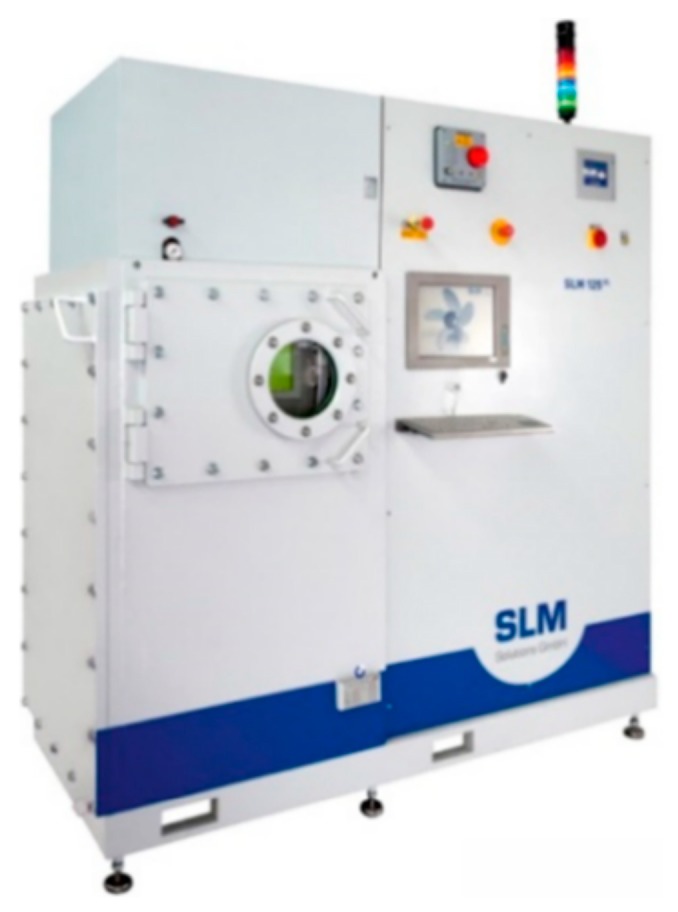
Modified SLM125HL system with overpressure chamber and build chamber reduction to 50 × 50 × 50 mm^3^.

The magnesium parts are manufactured using a modified SLM125HL system by SLM Solutions GmbH (Luebeck, Germany) with a 100 W continuous wave ytterbium fiber laser (see Figure 2). The modification of the machine was conducted to meet the advanced requirements of magnesium research and is therefore only useable for processing of magnesium powders to avoid contamination with other materials with regard to the manufacturing of medical implants. The laser has a wavelength of 1070 nm, a focus diameter of 70 µm, and a beam quality of M^2^ < 1.1. The L-PBF build jobs are conducted in an argon gas atmosphere.

Cylindrical specimens with different parameter combinations were manufactured for further investigation of the effect of different parameter settings and, therefore, different energy inputs during manufacturing. The parameters scanning speed, hatch distance, and layer size are varied. In addition, a double exposure was tested, which means that each layer of powder was coated and scanned twice, or in other words, the building platform was lowered only every second layer. The parameter settings are based on the results of preliminary investigations. The chosen parameter settings are given in Table 1. A laser power of 100 W was used.

#### 2.1.2. Titanium

**Figure 3:**
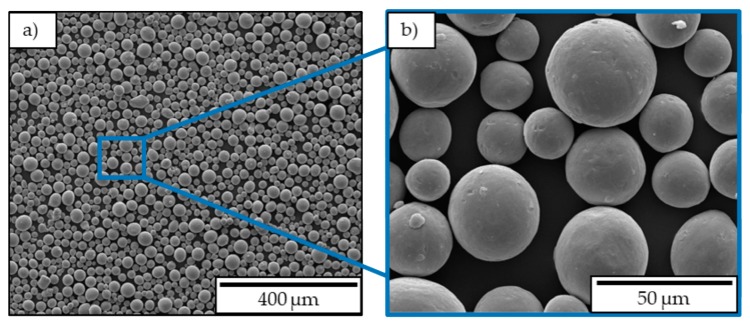
SEM images of Ti-6Al-4V powder: (**a**) ×250; (**b**) ×2000 magnification.

The material used for building the specimen is gas-atomized Ti-6Al-4V powder produced by Heraeus additive manufacturing GmbH with a particle size of 15 to 53 µm. A particle size analysis conducted by Heraeus additive manufacturing GmbH showed that 10% of the particles have a diameter smaller than 22.46 µm and 90% have a diameter smaller than 53.68 µm. The chemical analysis revealed that the actual value of the aluminum content is 6.07 wt % and the vanadium content is 3.94 wt %. SEM images, shown in Figure 3, were taken and analyzed to evaluate the particle geometry that appeared to be mostly spherical.

The manufacturing parameters (laser power P_L_, scanning speed v_S_, hatch distance d_H_) for building the test specimens are chosen according to the results of a preliminary parameter study. In this study, cubic specimen were built with different parameter settings using a design of experiments (DoE) approach. The evaluation of the results revealed three combinations of parameter settings that lead to high density parts and therefore promised favorable mechanical properties. The chosen parameter settings are shown in Table 2. Furthermore, a constant hatch distance of 60 µm and a layer size of 30 µm were implemented. Each layer was scanned only once.

For each parameter, setting cylindrical specimens were built and the industrial machine Lasertec 12 SLM (Realizer GmbH, Borchen, Germany) was used. It was equipped with an ytterbium fiber laser (continuous wave, single mode) operating at a wavelength of 1070 nm with a maximum power of 400 W, providing a laser beam with a beam quality factor of M^2^ = 1.05. The machine offers a minimum beam diameter of 35 µm and a building volume of 125 × 125 × 200 mm^3^. To avoid oxidation, the process chamber is filled by the inert gas argon. This way, it is possible to reach a minimum residual oxygen content of 0.13 to 0.15 wt %.

#### 2.1.3. Specimen Geometry

With regard to cyclic testing under stress ratio R = 10, i.e. compression–compression, no standard in case of the specimen geometry is available in literature. Solely German standard DIN 50106 [34] can be used to determine boundary conditions for the fatigue experiments. According to the German standard, the specimen geometry was chosen based on the following ratio between height (h_0_) and diameter (d_0_) for magnesium and titanium:

(1) $$1\leq \frac{h_{0}}{d_{0}}\leq 2$$

Therefore, the specimen diameter was set to 7 mm and height was set to 10.5 mm, enabling a ratio of 1.5. The compliance of the above-mentioned ratio is necessary to prevent experimental problems like bending or buckling of the specimens. Further on, specimens were manufactured upright, which corresponds to load direction. Before testing, the front surfaces of the specimens were sanded and polished to be mirror-like and plane-parallel.

### 2.2. Computer Tomography

Computer tomography (CT) provides a contactless and non-destructive insight into parts and structures. For both, magnesium and titanium specimens, porosity, and building defects are investigated. Therefore, the X-ray device nanotom 180 (GE Sensing and Inspection Technologies GmbH, Wunstorf, Germany (voxel size: 6 µm, voltage: 90 kV, current: 110 µA)) is used to achieve the 3D data which is processed and visualized by the software VGStudio max (my VGL2.2, Volume Graphics GmbH, Heidelberg, Germany). To calculate the relative density, the provided 3D data is converted to a stack of 2D images representing slices through the part in building direction. These images are analyzed through a python script written at Laser Zentrum Hannover e.V. to calculate the ratio between dark (pores) and light pixels (dense material). The slicing distance is chosen with regard to the smallest distance between layers or hatches, respectively. Accordingly, for magnesium, a distance of 15 µm (smallest hatch distance), and for titanium, a distance of 30 µm (layer thickness) was chosen. An important option offered by the evaluation script is the possibility to exclude detected pores with a diameter under or over a specified value from the density calculation. This way, the influence of imaging artifacts like image noise on the calculation can be minimized. Additionally, the script measures the pore cross-section areas and gives out the mean and maximum pore sizes for every slicing image. The CT analysis was conducted for three specimens of each batch. For each specimen, the mean value for the relative density was calculated. To verify the CT results, they were compared to density values achieved through image analysis of polished specimen sections.

### 2.3. Microstructure

Parts manufactured by L-PBF are known to show different microstructures compared to parts achieved through casting or forging. Mechanical, as well as corrosion, properties depend strongly on the phases and grain morphologies. Hence, it is important to investigate the achieved microstructure in the built specimens. The manufactured samples follow a metallographic preparation route consisting of cold embedding in an epoxy resin (Technovit Epox, Kulzer GmbH, Hanau, Germany), grinding, polishing (Tegramin, Struers ApS, Ballerup, Danemark) and etching in Nital 2% and Kroll’s reagent for magnesium and titanium, respectively. For microstructural analysis, light microscopy was employed (Aristomet, Leica Microsystems GmbH, Wetzlar, Germany), in which the samples were analyzed in their center and near the surface, in cross-sections parallel to the building direction. For fractographic analysis, the SEM Mira 3 XMU (Tescan, Brno, Czech Republic) was used.

### 2.4. Corrosion Tests

**Figure 4:**
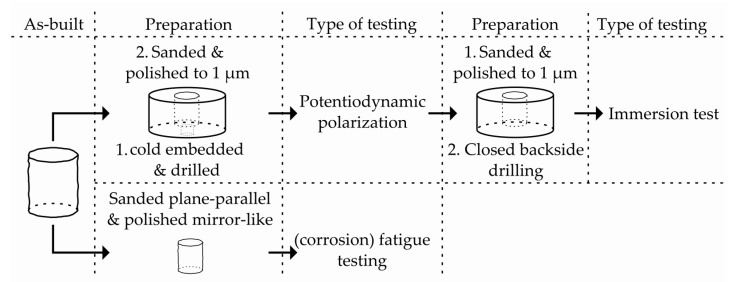
Overview of the sample preparation.

In order to characterize the corrosion behavior and to determine the corrosion rates, the immersion test was chosen in addition to the PDP. PDP only provides qualitatively comparable results, the reason for choosing the NDE having been described in the introduction. The immersion tests, on the other hand, provide quantitatively comparable results. For comparability of the tests, a simulated body fluid (SBF) according to Kokubo et al. [35] at 37 °C is used in both the PDP and the immersion tests (Table 3). The pH value is adjusted to 7.5 via 0.1 molar HCl. In order to obtain a direct correlation between PDP and immersion tests, the same samples were used for both test methods. The cylindrical specimens are cold embedded upright in an epoxy resin and sanded with grinding discs with grits of 800 to 2500 and polished to 6, 3, and 1 µm with a water-free diamond suspension. Before the immersion tests, the corrosion layer from the PDP is removed in the same way. For a first estimation of the corrosion behavior, polished surfaces are investigated instead of ‘as-built’ surfaces to eliminate the influence of surface roughness. A backside drilling through the embedding material enables the contacting for the electrochemical measurements. An overview of the sample preparations before the respective investigations is given in Figure 4.

#### 2.4.1. Potentiodynamic Polarization

**Figure 5:**
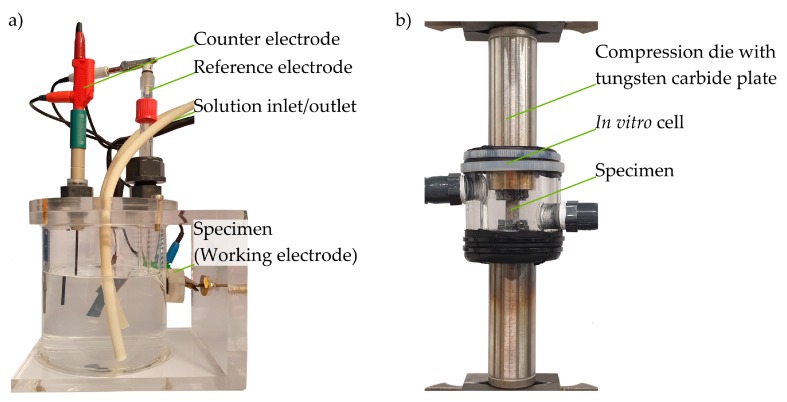
Experimental setup for (**a**) potentiodynamic polarization measurements; (**b**) corrosion fatigue tests.

A standard three-electrode system was used for this test method, and the test setup is shown in Figure 5a. The electrolyte was tempered via a hot plate with its control circuit and circulated via two pump circuits of a peristaltic pump so that a temperature of 37 °C is reached inside the corrosion cell. The inlet and outlet of the electrolyte were aligned in such a way that any influence on the electrochemical measurement was kept to a minimum. The embedded sample was pressed via an O-ring against an opening in the corrosion cell so that the polished surface was in contact with the electrolyte. For the three-electrode system, an Ag/AgCl electrode was used as a reference electrode and a graphite electrode as a counter electrode. The experimental setup, including the corrosion cell, was located in a Faraday cage, whereas the electrical devices (hot plate, peristaltic pump) were located outside the cage. Before carrying out the measurements, the open circuit potential (OCP) was measured for 30 min. Afterward, the sample was polarized with a potential feed of 0.8 mV s^−1^ within a potential range of ±300 mV. The Gamry potentiostat PCI4300 (Gamry Instruments Inc., Warminster, PA, USA) was used, as well as the corresponding Gamry Echem Analyst software, to evaluate the corrosion current density i_corr_ based on the Butler–Volmer equation.

#### 2.4.2. Immersion Tests

As described before, the same samples of the PDP were used, only the drilling on the back was closed. A double-walled corrosion cell was used, whereby distilled water is conveyed through the outer jacket by a thermostat so that a temperature of 37 °C is reached in the inner jacket. The sample was placed below a burette (total volume 10 mL, calibration marks at 0.02 mL) so that the hydrogen produced was collected by the burette funnel and the volume can be determined from the level inside the burette. Due to the high ratio of electrolyte volume to sample surface and the short test times, no saturation effects are expected. The tests are carried out until a constant corrosion rate is reached. For evaluation, the specific hydrogen volume is plotted over the immersion time. The corrosion rate is determined by the slope of the linear section of a compensation curve.

### 2.5. Corrosion Fatigue Tests

Based on the results of the corrosion tests on magnesium, two batches (batch A_Mg_ and B_Mg_) were chosen for the cyclic tests, which suggests the greatest differences in fatigue behavior. For titanium, one batch (batch R_Ti_) was selected for the corrosion fatigue tests, based on results from CT analysis. Constant amplitude tests (CAT) were carried out in SBF at 37 °C to simulate in vivo conditions and as a reference in air at room temperature (RT). The same electrolyte was used as was used in the corrosion tests. The investigations were carried out on a servo-hydraulic testing machine Schenck PC 63M with Instron 8800 controller (Instron, Norwood, MA, USA (F_max_ = ±45 kN)) with adapted compression dies (Inconel 602), as well as tungsten carbide plates mounted on the end faces. An in vitro corrosion cell was developed to enable a medial superposition by the SBF (Figure 5b). In order to reach a constant temperature of 37 °C and to prevent saturation effects inside the cell, the test setup was instrumented with a thermostat and a peristaltic pump. The minimum volume flow of the peristaltic pump and the internal diameter of the tubes used resulted in a fluid velocity of 0.003 m/s via continuity law. Thus, the flow velocity used is far below the mean aortic velocity of a human (1.12–1.32 m/s) [36], so that the influence of the velocity on the corrosion behavior is negligible. In order to prevent the specimens from galvanic corrosion, the compression dies, together with the tungsten, carbide plates were coated with a polyurethane lacquer.

CAT was performed at a stress ratio of R = 10 (compression–compression), a sinusoidal stress–time function, and a constant test frequency of f_Mg_ = 10 Hz and f_Ti_ = 5 Hz. Based on previous investigations, a lower frequency was selected for titanium to prevent a significant increase in temperature. The stress ratio is considered as the first estimation for an implant that is mainly loaded by compression. For further investigations, the experimental setup and the specimen geometry can be adapted for other applications and stress ratios. The tests were carried out until the final fracture of the specimen and a run out was defined with a maximum number of cycles of N_limit_ = 2 × 10^6^. Since the tests on the titanium specimens are only used as a reference to the biodegradable magnesium, the maximum number of cycles was adapted to magnesium, based on the results of the corrosion fatigue tests.

## 3. Results

### 3.1. Computer Tomography

**Figure 6:**
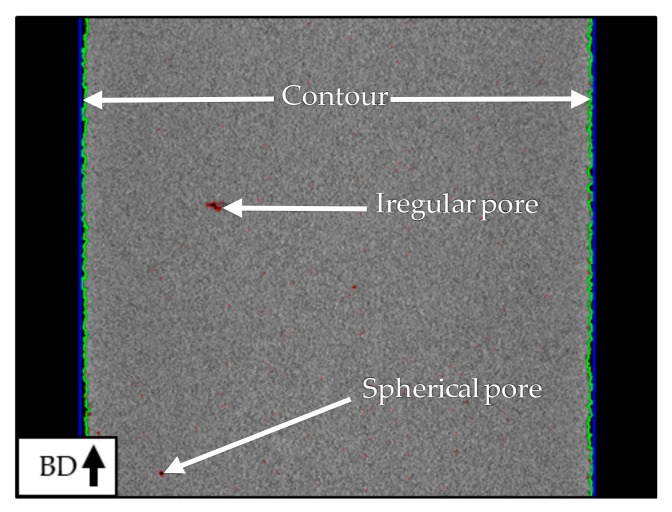
Computer tomography image of cylindrical titanium specimen.

All specimens showed mostly dense material on the CT images and a high relative density of over 99.8% in the subsequent analysis. Due to the high image noise, a suitable greyscale threshold had to be found and a threshold value of 65 (black = 0, white = 255) offered the best differentiation between pores and dense material. In addition, detected pores with a diameter of under 15 µm were identified to be imaging artifacts caused by image noise and therefore were excluded from the further analysis. Figure 6 shows an example of a CT image after the analysis. Pores are marked red and the contour is marked in light green. Regarding the individual pores, there generally are two types of defect geometries. On one hand, there are large and irregular pores, and on the other hand, there are small, spherical pores that also outnumber the irregular ones.

#### 3.1.1. Magnesium

**Figure 7:**
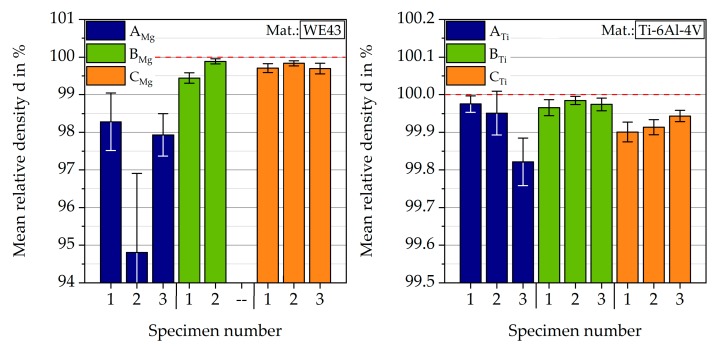
Mean relative densities of magnesium and titanium specimens.

Based on the computer tomography data, the mean relative density for three specimens for each batch was calculated (see Figure 7).

The CT dataset of one specimen from batch B_Mg_ was damaged, which led to only two calculated densities for batch B_Mg_. The second specimen within batch B_Mg_ with a smaller layer size of 30 µm reveals the highest mean relative density of 99.89%. This specimen also shows the smallest standard deviation of 0.07% relative density. A high mean density of 99.84 ± 0.07% was also detected for the second specimen of batch C_Mg_ with a double exposure build strategy. The second specimen of batch A_Mg_ leads to the lowest calculated mean relative density of 94.80%. For this specimen, the highest standard deviation of 2.11% relative density was found. The investigated specimens show an increasing standard deviation with decreasing mean relative density. To verify the results from CT, the relative density is also measured in cross-sections after metallographic preparation by the use of image analysis (the mean value is calculated of the three specimens for three cross-sections each). For batch B_Mg_ the similar relative densities with 99.89 ± 0.06% was measured. The relative density of batch C_Mg_ is with 99.84 ± 0.07%, slightly higher than the value received from the CT data. The relative density measured in the cross-sections of samples from batch A_Mg_ reveals a higher relative density of 97.13 ± 0.76%.

**Figure 8:**
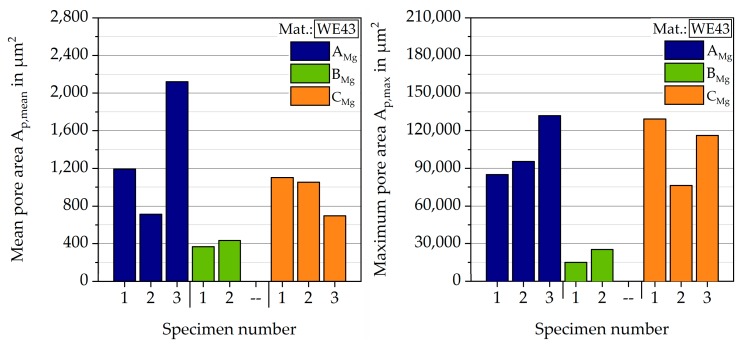
Mean and maximum pore area of magnesium specimens.

The calculated mean and maximum pore sizes are given in Figure 8. In general, specimens of batch B_Mg_ show the smallest mean pore area, between 350 and 450 µm^2^. For specimens from batch C_Mg_, mean pore areas between 650 and 1100 µm^2^ were detected. For batch A_Mg_, a high spreading was found, with mean pore areas between 700 and 2200 µm^2^. The specimens of batch B_Mg_ show the smallest pore areas with about 15,000 and 25,200 µm^2^. Specimens from batch A_Mg_ and C_Mg_ both lead to bigger pores, with calculated maximum pore areas between 76,000 and 131,900 µm^2^.

#### 3.1.2. Titanium

For all tested specimens, the mean relative density of all slices, as well as the standard deviation, were calculated, and are displayed in Figure 7. 

The highest mean value of 99.98% relative density was detected for batch B_Ti_. Additionally, this specimen also showed the smallest standard deviation of 0.01% relative density. A minimum value for the relative density of 99.82% and the maximum standard deviation of 0.06% were calculated for the third specimen of batch A_Ti_. Generally, an increasing standard deviation was observed for a decreasing relative density. Of all parameter settings, batch A_Ti_ led to the specimens with the lowest relative density and with the highest standard deviation of the relative density. As for magnesium, the CT results were compared to the relative density evaluated through microscope image analysis of polished specimen sections. These values were slightly lower with mean values of 99.06%, 99.21%, and 99.69% for the batches A_Ti_, B_Ti_, and C_Ti_, respectively. Nevertheless, it could be shown that high densities of over 99% can be achieved.

**Figure 9:**
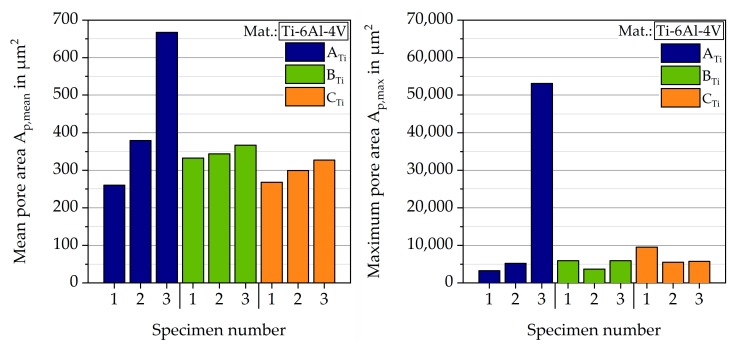
Mean and maximum pore area of titanium specimens.

In addition to the relative densities, the mean and the maximum pore size were calculated for each sample, displayed in Figure 9, respectively. Except for one specimen of batch A_Ti_, all specimens had a mean pore area between 250 and 400 µm^2^ and the biggest pore had an area of under 10,000 µm^2^. The one exceptional high value could, therefore, be regarded as an outlier.

Since Ti-6Al-4V is used here as a reference for the magnesium alloy and all three batches show similar microstructure and process-induced porosity, only specimens from batch B_Ti_, which have, by comparison, the highest relative density, were used for mechanical testing. They will be subsequently named as batch R_Ti_.

### 3.2. Microstructure

#### 3.2.1. Magnesium

**Figure 10:**
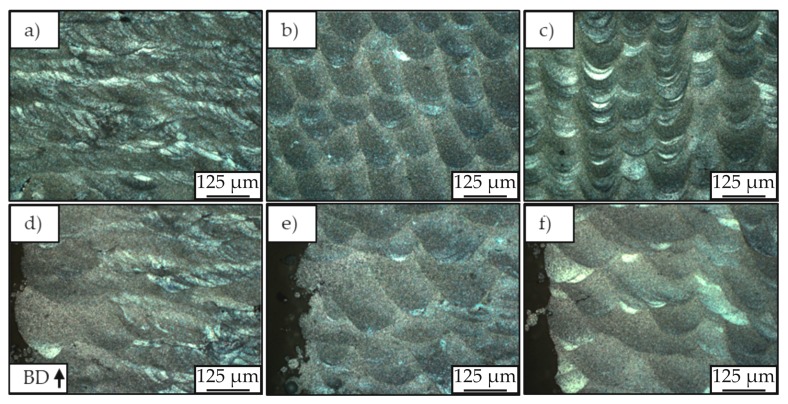
Light microscopy pictures of center (**a**–**c**) and surface (**d**–**f**) of samples in batch A_Mg_ (**a**,**d**), B_Mg_ (**b**,**e**) and C_Mg_ (**c**,**f**). Etchant: Nital 2%.

In lower magnifications of 200× (Figure 10), it is possible to verify that not only the general morphology of the solidified melting pools varies between the different batches, but also that all samples present a number of heterogeneities along its microstructure. While the cross-sections associated to batch A_Mg_ (a and d) show many small, single solidified melt pools, batch B_Mg_ (b and e) and C_Mg_ (c and f) present bigger and deeper melt pools, a consequence of lower scan speeds and, therefore, higher energy inputs. Batch C_Mg_, in turn, presents a melt pool superimposition without an overlapping zone, which increases the general energy input and is correlated to the double laser beam exposure. The aforementioned melt pool depths have been measured to be approximately 1, 5–6, and 3–4 times the layer size for batches A_Mg_, B_Mg_, and C_Mg_, respectively. The center of the cross-section of the samples belonging to batch A_Mg_ shows small single melt pools compared to B_Mg_ and C_Mg_. In contrast, the surface of the samples belonging to batch A_Mg_ shows greater melt pools. This is correlated with the decreased thermal conductivity of the surrounding powder bed, resulting in an accumulation of heat, whereas the higher thermal conductivity in the bulk material leads to smaller melt pools.

**Figure 11:**
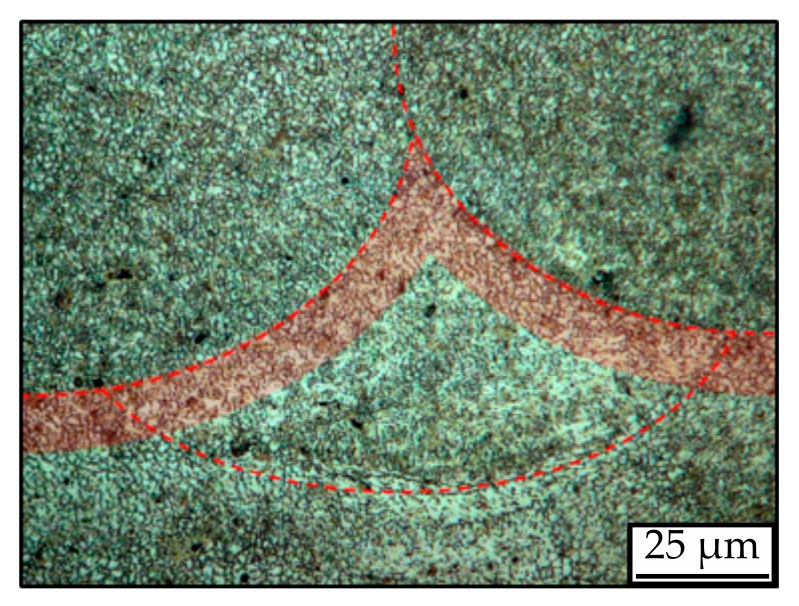
Heat affected zone of superimposition region of two neighboring melt pools in a sample of batch B_Mg_. Etchant: Nital 2%.

Higher magnifications of 1000× permit the estimation of the grain sizes associated to each batch. All conditions present very fine grains with diameters of a few micrometers, whose size seems to show little to no increase in the partially molten regions of the heat affected zone (Figure 11). Although the grain boundaries are weakly etched, a network of dark phases can be identified in such regions, which permits the identification of grain sizes.

**Figure 12:**
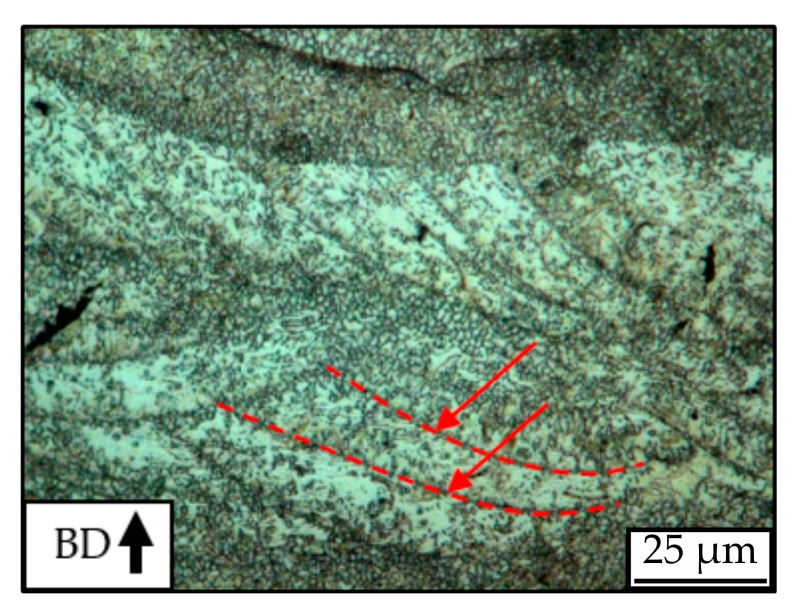
Region poor in intergranular intermetallic network, sample of batch A_Mg_. Etchant: Nital 2%.; red lines show interfacial precipitation.

Regions poor in such intergranular phase network are also identified, especially in samples of batch A_Mg_ (Figure 12). Such regions appear brighter in the light microscopy pictures, given their higher reflectivity when compared to densely precipitated regions. Precipitation is, however, still present in these areas, mostly in the interfacial region between the melt pool and the heat affected zone.

#### 3.2.2. Titanium

**Figure 13:**
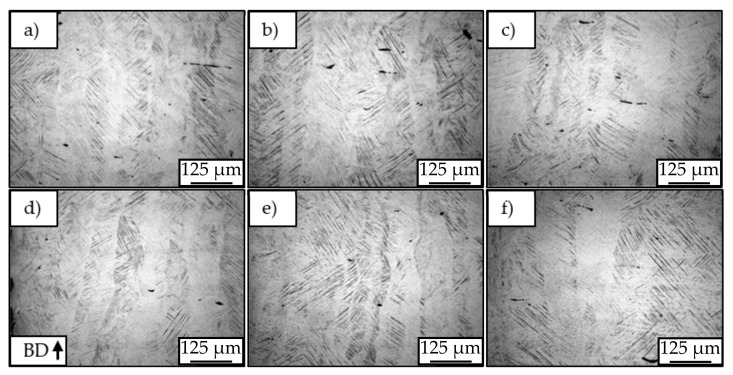
Light microscopy pictures of center (**a**–**c**) and left side surface (**d**–**f**) of batch A_Ti_ (**a**,**d**), B_Ti_ (**b**,**e**) and C_Ti_ (**c**,**f**). Etchant: Kroll’s reagent.

Other than for magnesium, the microstructural analysis of the titanium specimens did not reveal visible solidified melt pools or clear borders between the single layers. All batches led to a similar microstructure, as can be seen in Figure 13.

**Figure 14:**
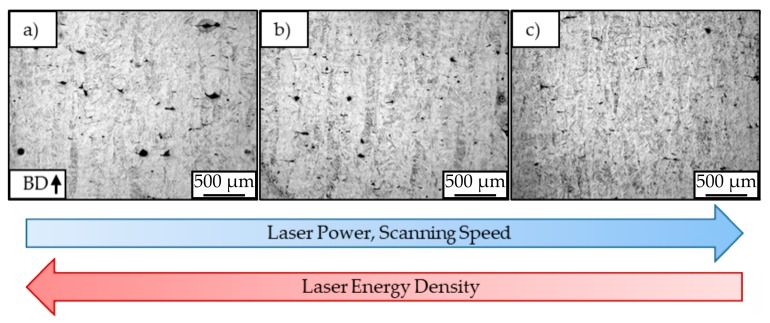
Microstructure for different batches A_Ti_ (**a**), B_Ti_ (**b**), and C_Ti_ (**c**).

For all specimens, the typical microstructure of L-PBF processed Ti-6Al-4V was observed that was also described in different literature [30,37,38]. As the microscopy pictures show, the microstructure exhibits elongated prior-β grains filled with fine α’-martensite laths of different lengths and widths. These martensite laths are oriented around 45° to the building direction. The measurement of the microscope images revealed a width of the prior-β grains up to around 300 µm. Near the surface (Figure 13d–f), the grains are slightly smaller and less oriented than in the center of the samples (a,b,c). The microscopy pictures with smaller magnifications (Figure 14) reveal a slight refinement of the microstructure with increasing laser power and scanning speed, due to the simultaneous increase of these parameters a decrease in laser energy input. Visible signs of the refined microstructure are a decrease in width of the prior-β grains and therefore a decrease in the length of the α’-martensite laths.

**Figure 15:**
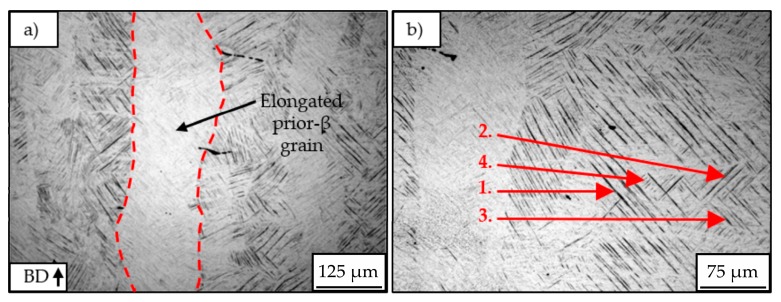
L-PBF typical acicular α’-martensite structure: (**a**) elongated prior-β grains, (**b**) different martensites (1. primary, 2. secondary, 3. tertiary, 4. quartic).

Figure 15 offers a closer look at the grain and martensite morphology. The grain boundaries are marked red exemplarily for one grain for better visibility (a). As described in [38,39], different types of α’-martensite can be recognized through their different lath sizes and orientations: Primary, secondary, tertiary, and quadric martensite (b).

### 3.3. Corrosion Tests

#### 3.3.1. Potentiodynamic Polarization

**Figure 16:**
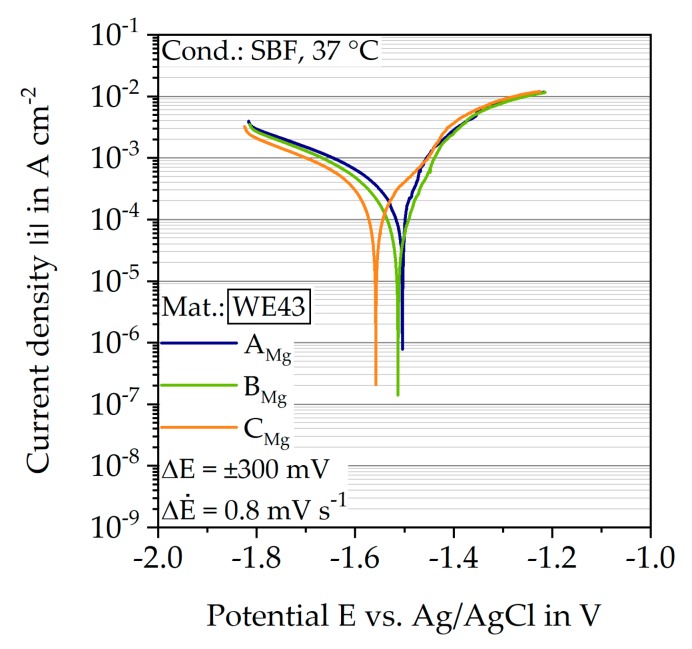
Results of the potentiodynamic polarizations measurements of the three batches in SBF, shown as Tafel plot.

The execution of PDP measurements is valid for a first estimation of the corrosion resistance and corrosion rate ${\dot{m}}_{\mathrm{corr}}$ by determining the corrosion potential E_corr_ and the corrosion current density i_corr_. Figure 16 shows the corresponding results as a Tafel plot so that the current density |i| is plotted semi-logarithmically over the potential E relative to an Ag/AgCl electrode. In addition, the respective characteristic values are listed in Table 4. The different batches show a qualitatively similar course and differ barely in their corrosion potentials E_corr_ (−1.50 V for batch A_Mg_, −1.56 V for batch C_Mg_). The anodic Tafel lines have a similar gradient and no passivation area is recognizable. The corrosion rates are calculated using i_corr_ based on Faraday’s law, whereby i_corr_ is determined using Gamry Echem Analyst software based on the Butler–Volmer equation. In contrast to the corrosion potentials, batch A_Mg_ had the highest and batch B_Mg_ had the lowest i_corr_, consequently ${\dot{m}}_{\mathrm{corr}}$ is calculated to 1.5∙10^3^ mg/(cm^2^ a) for batch A_Mg_ and 0.6∙10^3^ mg/(cm^2^ a) for batch B_Mg_.

#### 3.3.2. Immersion Tests

**Figure 17:**
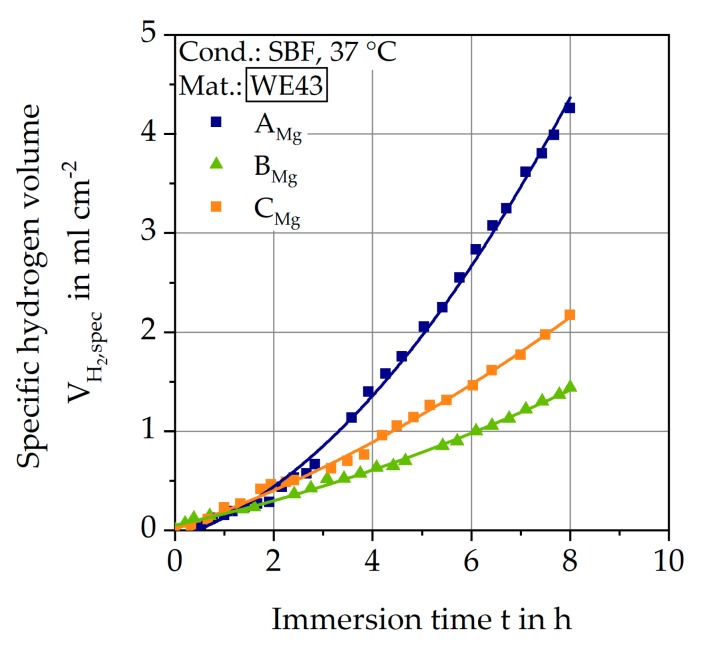
Results of immersion tests in SBF at 37 °C.

Figure 17 shows the results of the immersion tests of the three batches in SBF at 37 °C. The specific hydrogen formation $V_{H_{2},\mathrm{spec}}$ is plotted as a function of the immersion time t. For all three batches, a qualitatively similar, progressive course is recognizable. $V_{H_{2},\mathrm{spec}}$ increases linearly after a start interval of 3 to 4 h. Accordingly, a constant hydrogen formation rate occurs after this time, and this slope is used to calculate through a linear approximation ${\dot{m}}_{\mathrm{corr}}$ (Table 4). Batch A_Mg_ reached the highest value with 7.2∙10^3^ mg/(cm^2^ a) and batch B_Mg_ reached the lowest value with 2.2∙10^3^ mg/(cm^2^ a). Based on these results and the pore distributions of the CT scans, batches A_Mg_ and B_Mg_ are used for subsequent fatigue and corrosion fatigue tests. The different corrosion properties and pore distributions suggest the greatest differences in fatigue behavior.

### 3.4. Corrosion Fatigue Tests

**Figure 18:**
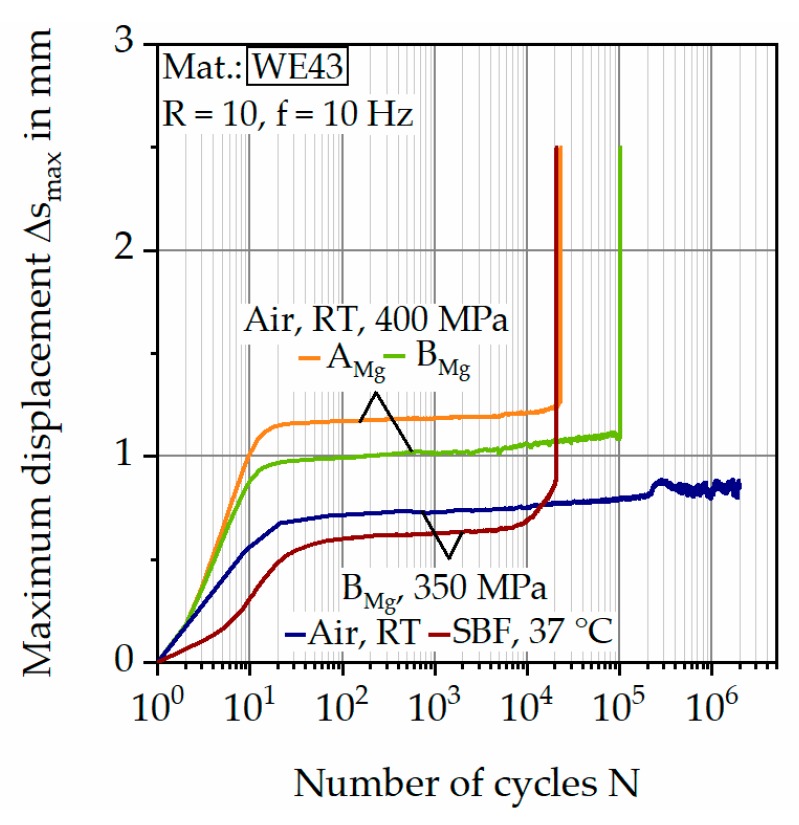
Δs_max_-N curves for different batches and testing conditions.

Figure 18 shows the results of the CAT in form of the material reaction with the maximum displacement Δs_max_ as a function of the number of cycles N in a semi-logarithmic plot, hence, the course within the first 100 cycles can be seen especially. 

Two parameter sets (batches and environmental conditions) were compared with otherwise constant parameters. A similar behavior within the first 40 cycles is recognizable for the four displayed curves, the respective curve is progressive and approaches a saturation value, followed by a plateau, in which the change of Δs_max_ is moderate. Both experiments in air (at 400 MPa) also show a similar course before failure occurs. The failure is announced only slightly by an increase of Δs_max_, consequently, the samples fail brittle. The initial deformation is greater for batch A_Mg_ and the failure occurs earlier. For the shown test in the SBF, a progressive path before failure is observed.

**Figure 19:**
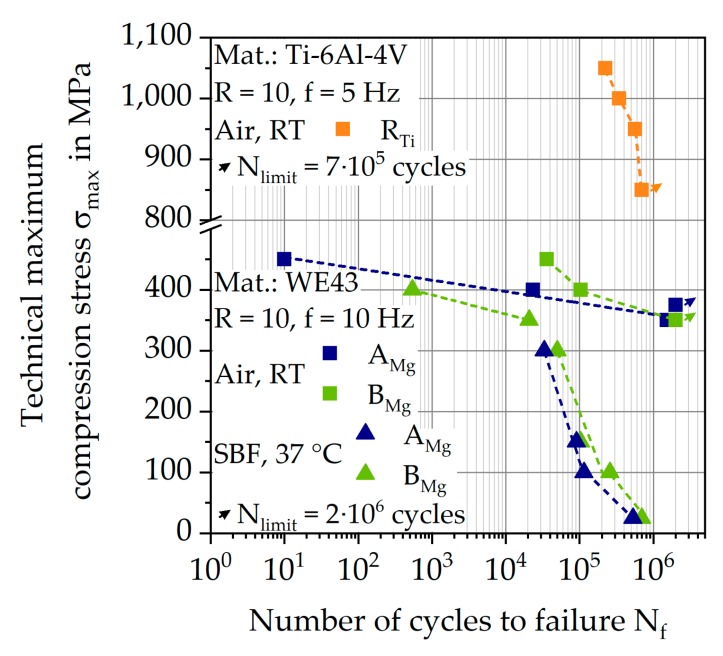
Trend S-N curves for batches A_Mg_ and B_Mg_ of magnesium and batch R_Ti_ of titanium at RT and in SBF at 37 °C.

The summarized results of the CAT of both batches in SBF at 37 °C and in air at RT are shown as trend S-N curves in Figure 19. For the tests in air, differences in the low cycle fatigue (LCF) range between the two batches are visible, for a technical maximum compression stress σ_max_ = 450 MPa batch A_Mg_ achieves a number of cycles at failure N_f_ = 10 cycles, whereas batch B_Mg_ achieves N_f_ = 35,979 cycles. In the high cycle fatigue (HCF) range, a similar trend can be seen for σ_max_ = 400 MPa, nevertheless, the run outs (N_limit_ = 2∙10^6^ cycles) for σ_max_ = 350 MPa (batch B_Mg_) and 375 MPa (batch A_Mg_) are achieved in a similar range of σ_max_. For both batches, the respective data points of the CAT in air can be approximated linearly. 

With regard to Ti-6Al-4V, a higher overall maximum compression stress σ_max_ can be achieved (Figure 19). Under a load of σ_max_ = 1050 MPa, a total number of cycles to failure N_f_ = 224,542 cycles was determined, which subsequently increases to N_f_ = 564,550 cycles under σ_max_ = 950 MPa, implicating the high strength of titanium in comparison to the magnesium alloy. A general approach for testing the corrosion fatigue properties of Ti-6Al-4V is to immerse specimens for a certain time, which can be up to several weeks in SBF [6,7], and subsequently test them. As can be seen in the investigations from Liu et al. [7], this can have little to no influence on the fatigue properties of Ti-6Al-4V in the HCF range. For enabling the same testing conditions for both materials, no fatigue tests under SBF were carried out for Ti-6Al-4V, since it can be assumed that the influence of corrosion on the fatigue properties of Ti-6Al-4V is negligible for short time experiments.

However, the results of magnesium alloy in SBF show different material behavior. In the LCF range of the CAT in SBF, batch B_Mg_ reaches N_f_ = 542 cycles at σ_max_ = 400 MPa, and 20,670 cycles at σ_max_ = 350 MPa. The trend S-N curve then bends, and N_f_ for both batches increases only slightly for decreasing σ_max_. Compared to σ_max_ = 300 MPa with N_f_ = 33,229 cycles for batch A_Mg_ and 49,503 cycles for batch B_Mg_, the number of cycles at failure for σ_max_ = 50 MPa only increases to N_f_ = 236,966 and 388,045 cycles, respectively. For the lowest technical maximum compression stress of σ_max_ = 25 MPa, the number of cycles to failure for batch B_Mg_ is N_f_ = 691,729 cycles. A run out could not be achieved for both batches, but the course in the HCF range seems to flatten out towards a higher number of cycles. For each σ_max_ of the CAT in SBF, batch B_Mg_ achieves a higher N_f_ than batch A_Mg_. Lower values for σ_max_ could not be accomplished regarding the calibration of the machine used. 

### 3.5. Fractography

**Figure 20:**
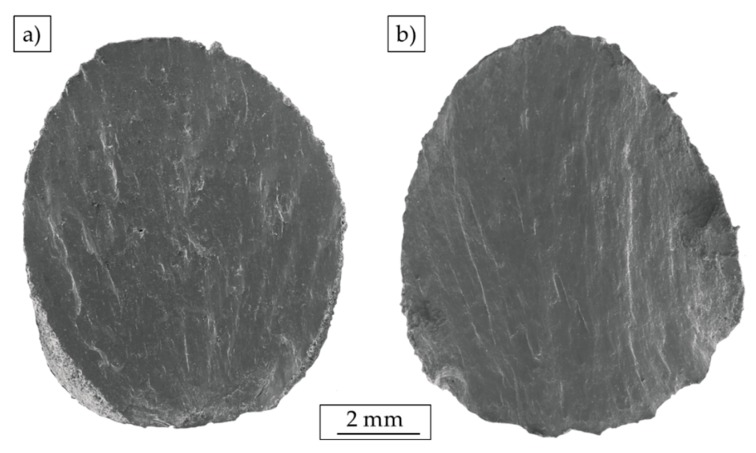
SEM images of fractured specimens tested at 400 MPa in air at RT: (**a**) batch A_Mg_ (N_f_ = 23,192 cycles) and (**b**) batch B_Mg_ (N_f_ = 102,848 cycles).

Results from the fractographic analysis are presented in Figure 20, whereas Figure 20a shows the fractured surface of batch A_Mg_ (σ_max_ = 400 MPa, N_f_ = 23,192 cycles) and Figure 20b shows the corresponding surface of batch B_Mg_ (σ_max_ = 400 MPa, N_f_ = 102,848 cycles). Both specimens were tested in air at RT. It can be seen that the specimens failed under an angle of 45° and fracture surfaces slipped off each other during failure, implicating a high degree of deformation. It can be noticed that crack initiation takes place at the surface, due to the presence of high surface roughness. However, primary crack initiation cannot be easily identified. This might be attributed to the increased surface roughness and the presence of notch-like defects at the surface of additively manufactured parts.

**Figure 21:**
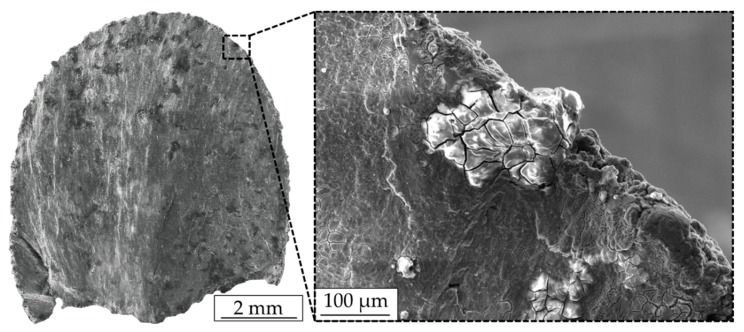
SEM images of an exemplary fractured surface of batch B_Mg_, tested in SBF at 37 °C.

An exemplary fractured surface of a specimen, tested in SBF at 37 °C, with a corresponding magnification of crack initiation, is depicted in Figure 21. As can be seen, failure occurred as well under an angle of 45°. However, corrosion pits can be found at the near-surface of the specimen, which locally support crack initiation. Nevertheless, even here, no single crack initiation can be detected.

## 4. Discussion

### 4.1. Computer Tomography

The CT analysis is facing the problem of high image noise. It partially could not be differentiated between very small real pores and dark pixels that were to lead back to the noise. Therefore, more pores, especially very small ones, were detected than actually exist in the specimens. This results in a decreased relative density and also a smaller mean pore size. On the other hand, pores appear smaller than they actually are on CT images. Accordingly, the real maximum pore size is bigger than the one evaluated in the CT analysis. The described problem could be solved partially by excluding the smallest detected pores from the calculation, as they were likely to be image artifacts. The differences and high variations in calculated relative density could also be shown by the comparison with conventionally achieved values for the relative density, which all were lower than the CT values for titanium and similar or higher for magnesium. The alternating values referred to the different methods to identify the relative densities, which can be attributed to measurements variations. The relative densities determined by image analysis have similar dependencies and are in the same range as those calculated from CT data. For a more precise evaluation of the relative density, a higher CT resolution and a reduction of image noise would be necessary. Besides the image quality, the positioning of the parts and the slightly varying scale of the resulting images also influences the analysis accuracy that should be kept in mind. It is therefore important to place all samples of an experimental run precisely in the same way inside the CT device. 

The different pore geometries observed are caused by different defect mechanisms. The small spherical pores are caused by trapped gas, often due to high energy input, whereas big and irregular pores indicate insufficient melting and a lack of fusion as a result of too low energy input or poor wettability.

The high standard deviation, especially for batch A_Mg_ and A_Ti_, means that in these specimens there are slices or layers with very big pores or a high number of pores, and on the other hand, slices or layers with a higher density. It could also be shown that the standard deviation is linked to the overall relative density of the specimen and increases with decreasing relative density. High standard deviation in part properties and as a result a lack of reproducibility are known challenges in additive manufacturing, and should therefore be taken into account when it comes to the planning and conduction of experiments.

### 4.2. Microstructure 

#### 4.2.1. Magnesium

The fine-grained microstructure of the alloy is expected due to the fast cooling of the melting pools in additive manufacturing [40]. In addition, for L-BPF-processed magnesium alloys, it has been reported that it possibly undergoes grain boundary locking via Zener pinning mechanisms associated to Y-rich phases in the intergranular region [41,42]. This tends to reduce the grain growth associated to local recrystallization in the partially molten regions of the heat affected zone, thus maintaining the fine cellular-dendritic grains along the alloy microstructure, as verified in this study. 

This grain refinement is expected to generally increase the mechanical properties like ultimate tensile and yield strength of the components through the Hall–Petch effect of inhibiting the motion of dislocations [43,44]. Nevertheless, Qin et al. argue that this is not expected to be a major effect for magnesium alloys [45]. Moreover, fine-grained microstructures are described as bringing higher corrosion resistance to magnesium systems, since the finer grains would be prone to produce more uniform corrosion products, a denser passivation layer and, ultimately, slow and homogeneous corrosion [44,46,47].

#### 4.2.2. Titanium

Titanium and corresponding alloys have a relatively poor thermal conductivity. The alloy Ti-6Al-4V processed in this work has a thermal conductivity of 7 W/(m K) at room temperature [48], leading to an accumulation of heat in the built part. Due to this, the already solidified material is remelted several times and the heat affected zone extends over several previous layers. As a result, the grains consisting of the high temperature β-phase grow across the layers and no single melt pools or layer boundaries are visible. The β-grains grow in the opposite direction of the heat flow. For the largest part of the volume, this means that the grains grow in the building direction. Near the lateral surfaces, the grain growth is less oriented due to the different and more complex heat flow in the cross region between the dense material and unmelted powder.

The width of the β-grains depends on the chosen parameter values, and therefore the laser energy input. Higher speeds lead to increased cooling rates so that there is less time for the grains to grow. As a result, a finer grain structure is received. On the other hand, a higher energy input provides more thermal energy for the grain growths, leading to a coarser microstructure.

In L-PBF, high cooling rates of 104–106 K/s are achieved [49,50], preventing the formation of the equilibrium α+β microstructure. Instead, the β-phase is diffusionless, transformed into α’-martensite that fills the prior-β grains. Thereby, the amount of the martensite phase depends on the cooling rate, which has to be sufficiently high, and the build temperature, which needs to be below the martensite start temperature [30]. As described by [38], a hierarchical structure of different types of martensite (primary, secondary, tertiary, quartic) was observed. This morphology can be led back to the cyclic reheating and remelting that is typical for the L-PBF process and can be influenced by varying the processing parameters [30].

Similar to magnesium, the microstructure, and therefore the grain size as well as the element distribution, influence the corrosion resistance and fatigue crack resistance, as stated by different literature [51,52,53].

### 4.3. Corrosion Behavior 

In general, the results of the two methods (PDP and immersion test) for determining the corrosion rate ${\dot{m}}_{\mathrm{corr}}$ of the three batches show quantitatively different results (Table 4). For all three batches, ${\dot{m}}_{\mathrm{corr}}$ determined by the immersion tests are higher. However, this coincides with literature data, since, apart from the problem of the NDE, the origin of which is still being discussed [17], this leads to lower corrosion current densities i_corr_, measured with comparable methods. In addition to the test parameters (potential range, potential feed, etc.), the evaluation method of i_corr_ in the Tafel plot also influences the calculated ${\dot{m}}_{\mathrm{corr}}$, leading to high standard deviations within this method [54]. Other studies exhibited values of i_corr_ in a wide range between 11.9 and 409 µA/cm^2^ [33,55,56] for different treatment methods of the WE43 alloy so that the results of this study (160.6 ≤ i_corr_ ≤ 380.3 µA/cm^2^) are within this range. The magnesium alloy WE43 is nominally a wrought material, but it is difficult to compare the corrosion properties because different treatment processes, especially heat treatments and other electrolytes, are used for these alloys [57]. Chu and Marquis determined lower values of i_corr_ = 70 and 85 µA/cm^2^, respectively, in PDP measurements, however, in contrast to the present study, a 3.5% NaCl solution was used [58]. Furthermore, for both test methods, the surfaces are polished at the beginning of the test. Due to the longer duration of the immersion tests, this is not continuously given within the test, so that an increased corroded surface area, and thus also an increased hydrogen formation, occurs. Nevertheless, the results prove that a qualitative evaluation of ${\dot{m}}_{\mathrm{corr}}$ based on PDP is possible and thus a statement about different material states can be made in a time-saving manner. A comparison with in vivo corrosion rates is difficult. On one hand, there are generally less in vivo results, on the other hand, Martinez Sanchez et al. showed in a review article that the corrosion rates in vitro exceed the results in vivo, and within one material, strong variations occur due to different in vitro testing conditions. For the alloy WE43, the average in vitro corrosion rate is 1.6 times higher than the in vivo value, with immersion tests executed with a SBF, Earle’s balanced salt solution, and minimum essential medium showing the best correlations to in vivo tests [59].

### 4.4. Fatigue Behavior 

For the two batches tested in fatigue and corrosion fatigue tests, there are clear differences, especially in the LCF range in air at RT. In the HCF range batch B_Mg_ achieves a run out at σ_max_ = 350 MPa, whereas batch A_Mg_ has a number of cycles to failure N_f_ = 1.52∙10^6^ cycles. These differences are mainly due to the different pore areas, as can be seen in Figure 8.

Due to the additional superposition through the SBF, the fatigue life of both batches decreases drastically. The bend in the trend S-N curve in the range 350 MPa ≤ σ_max_ < 250 MPa for batch B_Mg_ can probably be explained by the increased exposure time to the electrolyte and the resulting corrosion. The strongly decreasing slope for σ_max_ ≤ 250 MPa can thus also be justified by the increasing exposure time. Nevertheless, it should be noted that due to corrosion, the cross-section of the specimens decreases sharply with increasing test duration and thus the true stress increases. An example of this is the cross-section of one sample from batch B_Mg_ (σ_max_ = 25 MPa, N_f_ = 691,729 cycles). After the test, the cross-section was reduced to A ≈ 28 mm^2^ (from A_0_ = 40.9 mm^2^), so that based on the initial force a true stress of σ_max,true_ ≈ 36.4 MPa was reached at the end of the test. Both batches show very similar trend S-N curves for the tests in SBF, the course is in a good approximation parallel and they achieve a similar number of cycles to failure for the same stress levels. Based on porosity and corrosion rates, batch B_Mg_ also seems to have superior properties for corrosion fatigue, however, the general corrosive influence seems to predominate. Another possible explanation for the drastic decrease in fatigue strength might be notch-like defects at the surface, which result from plate-pile stacking defects and partially melted powder particles sticking on the specimen surface [60]. This can lead to stress corrosion, which was also observed by Bian et al. [25]. As can be seen in the results from the fractographic analysis, multiple crack initiation sites are present, resulting in the formation of several corrosion pits at the same time. With regard to available literature, a comparison with other data is difficult, due to various reasons. On one hand, there are generally few studies on the corrosion fatigue behavior of magnesium alloys in body-like fluids; on the other hand, both other magnesium alloys and test conditions were used in the existing studies. Studies on the corrosion fatigue behavior of additively manufactured magnesium alloys are not known to the authors. Zhao et al. investigated the influence of a precorrosion with different immersion times on the fatigue behavior [61]. Although this showed an influence of the precorrosion, a superimposed corrosive mechanical load nevertheless leads to far different damage mechanisms. Recent studies on corrosion fatigue behavior used a stress ratio of R = −1 which deviates from these tests, and also a test frequency of f = 10 Hz [24,25,62]. The common result of these studies was a strong decrease of the fatigue properties in body-like fluids compared to the reference in air, as well as a change of the failure mechanism. In air, crack initiation was caused by structural or mechanical defects, whereas in the electrolyte, initiation was caused by corrosion pits and thus drastically reduced the service life. Jafari et al. investigated the fatigue behavior of Mg-1Zn-0.3Ca for two extrusion temperatures. For both material states, the fatigue strength in air (N_limit_ = 10^7^ cycles) decreased from 106 and 81 MPa, respectively, to approximately 78 MPa for N_limit_ = 5∙10^5^ cycles. Despite similar properties in the PDP measurements, a stronger decrease of the corrosion fatigue properties in comparison to the reference condition in air was observed for the lower extrusion temperature [24]. For the calcium-containing alloys, Mg-2Zn-0.2Ca and Mg-1Ca, similar ratios between fatigue strength and corrosion fatigue strength could be determined for N_limit_ = 4∙10^6^ cycles. Thus, the values decreased from approximately 90 MPa to 70 and 68 MPa, respectively. A further finding of this study was the increase of ${\dot{m}}_{\mathrm{corr}}$ due to dynamic flow conditions in comparison to static fluids [25], which could also be proven by Levesque et al. [63]. Gu et al. also investigated the magnesium alloy WE43 and were able to determine a corrosion fatigue strength of 40 MPa for N_limit_ = 10^7^ cycles, as well as a stress ratio of R = −1 through tests in a SBF [52]. Jafari et al. refer the determined corrosion fatigue strength to a number of cycles; N = 10^5^ cycles, which, according to Taylor et al. is the fatigue limit for human bone at 23–30 MPa [63]. Such a number of cycles are achieved for magnesium alloy WE43 at σ_max_ = 150 MPa (batch B_Mg_) or 100 MPa (batch A_Mg_), so that there is sufficient fatigue strength in relation to Taylor et al. A limitation of this investigation is the deviation from the orthopedically maximum load frequency of 3 Hz [14], and the influence of a higher frequency has to be clarified. Furthermore, the used frequency f_Mg_ = 10 Hz reduces the test time and, consequently, also the exposure time to the electrolyte.

## 5. Conclusions and Outlook

Within the scope of this study, corrosion and corrosion fatigue tests were performed on the magnesium alloy WE43, and for reference purposes, on the titanium alloy Ti-6Al-4V to characterize the influence of the manufacturing parameters used in the laser powder bed fusion (L-PBF) process. Based on these results, the following conclusions can be drawn:

For magnesium, in particular, a strong dependence on the pore distribution and size as well as the microstructure was discernible for the selected manufacturing parameters. These properties hardly differed for titanium, due to chosen parameter combinations with very similar energy densities, consequently, only one parameter set was used for titanium for further investigations.

The corrosion rates, determined by the potentiodynamic polarization and immersion tests, also showed a dependence on the manufacturing parameters, which was attributed to the different microstructures. The corrosion rates determined by potentiodynamic polarization were below the values from the immersion tests. Furthermore, strong variations within one parameter set were measured. Nevertheless, a time-efficient estimation of the corrosion rates of different material states on the basis of potentiodynamic polarization is possible.

Based on the porosity, microstructure, and corrosion behavior, differences in fatigue and corrosion fatigue behavior could also be determined. For the reference tests in air, major differences were found between the two magnesium batches tested in the low cycle fatigue range. For the corrosion fatigue tests in SBF, there were continuous differences which, however, have to be evaluated as low, so that the general corrosion influence on the mechanical properties seems to be dominant here. Especially for increasing test durations, the mechanical fatigue properties decrease drastically, so that the trend S-N curve is bent. Based on the high surface roughness, primary crack initiation is difficult to determine, since multiple crack initiation is present, which locally supports corrosion. The reference alloy Ti-6Al-4V shows a higher overall strength in the fatigue tests.

Based on these findings, further studies should determine the exact influences of the individual manufacturing parameters in the L-PBF process on the macro- and microstructure, and thus on the corrosion and corrosion fatigue properties. Furthermore, the variations in the determination of corrosion rates must be minimized by a standardized procedure. An adaptation of the test load and thus of the test stress in the single-stage tests on the basis of the decreasing cross-section, so that tests are carried out at a constant true stress, is useful for understanding the influence of the macrostructure and microstructure on the fatigue properties.

## Figures and Tables

**Table 1:** Parameter settings for magnesium specimens.

Processing Parameter	Batch A_Mg_	Batch B_Mg_	Batch C_Mg_
Scanning speed in mm/s	1000	300	450
Hatch distance in µm	15	110	100
Exposure	single	single	double
Layer size in µm	50	30	50

**Table 2:** Parameter settings for titanium specimens.

Processing Parameter	Batch A_Ti_	Batch B_Ti_	Batch C_Ti_
Laser power in W	100	115	130
Scanning speed in mm/s	1000	1200	1400

**Table 3:** Ion concentration of the used simulated body fluid according to [35].

Ion Concentration in mmol/L							
**Simulated Body Fluid (SBF)**	**Na^+^**	**K^+^**	**Ca^2+^**	**Mg^2+^**	**(HCO_3_)^−^**	**Cl^−^**	**(HPO_4_)^2−^**
	142.0	5.0	2.5	1.5	4.2	147.8	1.0

**Table 4:** Characteristic values of the potentiodynamic polarization and immersion tests.

		${\dot{m}}_{\mathrm{corr}}$ in mg cm^−2^ a^−1^	
Batch	E_corr_ in V	PDP	Immersion
A_Mg_	−1.50	1.5∙10^3^	7.2∙10^3^
B_Mg_	−1.51	0.6∙10^3^	2.2∙10^3^
C_Mg_	−1.56	1.1∙10^3^	3.1∙10^3^

## References

[1] Arsiwala A., Desai P., Patravale V. (2014). Recent advances in micro/nanoscale biomedical implants. J. Control Release.

[2] Wilson J. (2018). Metallic Biomaterials. Fundamental Biomaterials.

[3] Murr L.E., Gaytan S.M., Medina F., Martinez E., Martinez J.L., Hernandez D.H., Machado B.I., Ramirez D.A., Wicker R.B. (2010). Characterization of Ti–6Al–4V open cellular foams fabricated by additive manufacturing using electron beam melting. Mater. Sci. Eng. A.

[4] Dallago M., Fontanari V., Torresani E., Leoni M., Pederzolli C., Potrich C., Benedetti M. (2018). Fatigue and biological properties of Ti-6Al-4V ELI cellular structures with variously arranged cubic cells made by selective laser melting. J. Mech. Behav. Biomed. Mater..

[5] Singh R., Lee P.D., Dashwood R.J., Lindley T.C. (2010). Titanium foams for biomedical applications: A review. Mater. Technol..

[6] Doi K., Miyabe S., Tsuchiya H., Fujimoto S. (2016). Degradation of Ti-6Al-4V alloy under cyclic loading in a simulated body environment with cell culturing. J. Mech. Behav. Biomed. Mater..

[7] Liu Y.J., Ouyang Q.L., Tian R.H., Wang Q.Y. (2009). Fatigue Properties of Ti-6Al-4V Subjected to Simulated Body Fluid. Struct. Longev..

[8] Orlov D., Joshi V., Solanki K.N., Neelameggham N.R. (2018). Magnesium Technology.

[9] Saris N.-E.L., Mervaala E., Karppanen H., Khawaja J.A., Lewenstam A. (2000). Magnesium—An update on physiological, clinical and analytical aspects. Clin. Chim. Acta.

[10] Staiger M.P., Pietak A.M., Huadmai J., Dias G. (2006). Magnesium and its alloys as orthopedic biomaterials: A review. Biomaterials.

[11] Jung O., Smeets R., Porchetta D., Kopp A., Ptock C., Müller U., Heiland M., Schwade M., Behr B., Kröger N. (2015). Optimized in vitro procedure for assessing the cytocompatibility of magnesium-based biomaterials. Acta Biomater..

[12] Jung O., Smeets R., Kopp A., Porchetta D., Hiester P., Heiland M., Friedrich R.E., Precht C., Hanken H., Gröbe A. (2016). PEO-generated Surfaces Support Attachment and Growth of Cells In Vitro with No Additional Benefit for Micro-roughness in Sa (0.2–4 µm). In Vivo.

[13] Li Z., Gu X., Lou S., Zheng Y. (2008). The development of binary Mg-Ca alloys for use as biodegradable materials within bone. Biomaterials.

[14] Singh Raman R.K., Jafari S., Harandi S.E. (2015). Corrosion fatigue fracture of magnesium alloys in bioimplant applications: A review. Eng. Fract. Mech..

[15] Kannan M.B., Raman R.K.S. (2008). In vitro degradation and mechanical integrity of calcium-containing magnesium alloys in modified-simulated body fluid. Biomaterials.

[16] Zander D., Schnatterer C. (2015). The influence of manufacturing processes on the microstructure and corrosion of the AZ91D magnesium alloy evaluated using a computational image analysis. Corros. Sci..

[17] Frankel G.S., Fajardo S., Lynch B.M. (2015). Introductory lecture on corrosion chemistry: A focus on anodic hydrogen evolution on Al and Mg. Faraday Discuss..

[18] Höche D., Blawert C., Lamaka S.V., Scharnagl N., Mendis C., Zheludkevich M.L. (2016). The effect of iron re-deposition on the corrosion of impurity-containing magnesium. Phys. Chem Chem Phys..

[19] Birbilis N., King A.D., Thomas S., Frankel G.S., Scully J.R. (2014). Evidence for enhanced catalytic activity of magnesium arising from anodic dissolution. Electrochim. Acta.

[20] Chen C., Liu T., Lv C., Lu L., Luo D. (2012). Study on cyclic deformation behavior of extruded Mg–3Al–1Zn alloy. Mater. Sci. Eng. A.

[21] Feng C.Y., Wang X.G., Tang P.H. (2012). Research on Low Cycle Fatigue Behavior and Life Prediction of Magnesium Alloy AZ91D. Adv. Mater. Res..

[22] Patel H.A., Rashidi N., Chen D.L., Bhole S.D., Luo A.A. (2012). Cyclic deformation behavior of a super-vacuum die cast magnesium alloy. Mater. Sci. Eng. A.

[23] Jafari S., Singh Raman R.K., Davies C.H.J. (2015). Corrosion fatigue of a magnesium alloy in modified simulated body fluid. Eng. Fract. Mech..

[24] Jafari S., Raman R.K.S., Davies C.H.J., Hofstetter J., Uggowitzer P.J., Löffler J.F. (2017). Stress corrosion cracking and corrosion fatigue characterisation of MgZn1Ca0.3 (ZX10) in a simulated physiological environment. J. Mech. Behav. Biomed. Mater..

[25] Bian D., Zhou W., Liu Y., Li N., Zheng Y., Sun Z. (2016). Fatigue behaviors of HP-Mg, Mg-Ca and Mg-Zn-Ca biodegradable metals in air and simulated body fluid. Acta Biomater.

[26] Krujatz F., Lode A., Seidel J., Bley T., Gelinsky M., Steingroewer J. (2017). Additive Biotech-Chances, challenges, and recent applications of additive manufacturing technologies in biotechnology. New Biotechnol..

[27] Singh S., Ramakrishna S. (2017). Biomedical applications of additive manufacturing: Present and future. Curr. Opin. Biomed. Eng..

[28] Tan X.P., Tan Y.J., Chow C.S.L., Tor S.B., Yeong W.Y. (2017). Metallic powder-bed based 3D printing of cellular scaffolds for orthopaedic implants: A state-of-the-art review on manufacturing, topological design, mechanical properties and biocompatibility. Mater. Sci. Eng. C Mater. Biol. Appl..

[29] Emmelmann C., Sander P., Kranz J., Wycisk E. (2011). Laser Additive Manufacturing and Bionics: Redefining Lightweight Design. Phys. Procedia.

[30] Liu S., Shin Y.C. (2019). Additive manufacturing of Ti6Al4V alloy: A review. Mater. Des..

[31] Wysocki B., Maj P., Sitek R., Buhagiar J., Kurzydłowski K., Święszkowski W. (2017). Laser and Electron Beam Additive Manufacturing Methods of Fabricating Titanium Bone Implants. Appl. Sci..

[32] Tammas-Williams S., Zhao H., Léonard F., Derguti F., Todd I., Prangnell P.B. (2015). XCT analysis of the influence of melt strategies on defect population in Ti–6Al–4V components manufactured by Selective Electron Beam Melting. Mater. Charact..

[33] Liu D., Yang D., Li X., Hu S. (2019). Mechanical properties, corrosion resistance and biocompatibilities of degradable Mg-RE alloys: A review. J. Mater. Res. Technol..

[34] (2016). DIN 50106–Testing of Metallic Materials–Compression Test at Room Temperature.

[35] Kokubo T., Kushitani H., Sakka S. (1990). Solutions able to reproduce in vivo surface-structure changes in bioactive glass-ceramic A-W3. J. Biomed. Mater. Res..

[36] Voges I., Jerosch-Herold M., Hedderich J., Pardun E., Hart C., Gabbert D.D., Hansen J.H., Petko C., Kramer H.-H., Rickers C. (2012). Normal values of aortic dimensions, distensibility, and pulse wave velocity in children and young adults: A cross-sectional study. J. Cardiovasc. Magn. Reson..

[37] Hao Y.-L., Li S.-J., Yang R. (2016). Biomedical titanium alloys and their additive manufacturing. Rare Met..

[38] Yang J., Yu H., Yin J., Gao M., Wang Z., Zeng X. (2016). Formation and control of martensite in Ti-6Al-4V alloy produced by selective laser melting. Mater. Des..

[39] Agius D., Kourousis K., Wallbrink C. (2018). A Review of the As-Built SLM Ti-6Al-4V Mechanical Properties towards Achieving Fatigue Resistant Designs. Metals.

[40] Bourell D., Kruth J.P., Leu M., Levy G., Rosen D., Beese A.M., Clare A. (2017). Materials for additive manufacturing. Cirp. Ann..

[41] Zumdick N.A., Jauer L., Kersting L.C., Kutz T.N., Schleifenbaum J.H., Zander D. (2019). Additive manufactured WE43 magnesium: A comparative study of the microstructure and mechanical properties with those of powder extruded and as-cast WE43. Mater. Charact..

[42] Luo K., Zhang L., Wu G., Liu W., Ding W. (2019). Effect of Y and Gd content on the microstructure and mechanical properties of Mg–Y–RE alloys. J. Magnes. Alloy..

[43] Jiang H.S., Zheng M.Y., Qiao X.G., Wu K., Peng Q.Y., Yang S.H., Yuan Y.H., Luo J.H. (2017). Microstructure and mechanical properties of WE43 magnesium alloy fabricated by direct-chill casting. Mater. Sci. Eng. A.

[44] Qin Y., Wen P., Guo H., Xia D., Zheng Y., Jauer L., Poprawe R., Voshage M., Schleifenbaum J.H. (2019). Additive manufacturing of biodegradable metals: Current research status and future perspectives. Acta Biomater..

[45] Xiang C., Gupta N., Coelho P., Cho K. (2018). Effect of microstructure on tensile and compressive behavior of WE43 alloy in as cast and heat treated conditions. Mater. Sci. Eng. A.

[46] Soderlind J., Cihova M., Schäublin R., Risbud S., Löffler J.F. (2019). Towards refining microstructures of biodegradable magnesium alloy WE43 by spark plasma sintering. Acta Biomater..

[47] Sing S.L., An J., Yeong W.Y., Wiria F.E. (2016). Laser and electron-beam powder-bed additive manufacturing of metallic implants: A review on processes, materials and designs. J. Orthop. Res..

[48] Parry L., Ashcroft I.A., Wildman R.D. (2016). Understanding the effect of laser scan strategy on residual stress in selective laser melting through thermo-mechanical simulation. Addit. Manuf..

[49] Gokuldoss P.K., Kolla S., Eckert J. (2017). Additive Manufacturing Processes: Selective Laser Melting, Electron Beam Melting and Binder Jetting-Selection Guidelines. Materials.

[50] Qiu C., Kindi M.A., Aladawi A.S., Hatmi I.A. (2018). A comprehensive study on microstructure and tensile behaviour of a selectively laser melted stainless steel. Sci. Rep..

[51] Lee E., Vasudevan A., Sadananda K. (2005). Effects of various environments on fatigue crack growth in Laser formed and IM Ti–6Al–4V alloys. Int. J. Fatigue.

[52] Bache M.R., Evans W.J. (2001). The fatigue crack propagation resistance of Ti–6Al–4V under aqueous saline environments. Int. J. Fatigue.

[53] Fleck C., Eifler D. (2010). Corrosion, fatigue and corrosion fatigue behaviour of metal implant materials, especially titanium alloys. Int. J. Fatigue.

[54] Kirkland N.T., Birbilis N., Staiger M.P. (2012). Assessing the corrosion of biodegradable magnesium implants: A critical review of current methodologies and their limitations. Acta Biomater..

[55] Davenport A.J., Padovani C., Connolly B.J., Stevens N.P.C., Beale T.A.W., Groso A., Stampanoni M. (2007). Synchrotron X-Ray Microtomography Study of the Role of Y in Corrosion of Magnesium Alloy WE43. Electrochem. Solid State Lett..

[56] Jin W., Wu G., Feng H., Wang W., Zhang X., Chu P.K. (2015). Improvement of corrosion resistance and biocompatibility of rare-earth WE43 magnesium alloy by neodymium self-ion implantation. Corros. Sci..

[57] Ninlachart J., Karmiol Z., Chidambaram D., Raja K.S. (2017). Effect of heat treatment conditions on the passivation behavior of WE43C Mg-Y-Nd alloy in chloride containing alkaline environments. J. Magnes. Alloys.

[58] Chu P.-W., Marquis E.A. (2015). Linking the microstructure of a heat-treated WE43 Mg alloy with its corrosion behavior. Corros. Sci..

[59] Martinez Sanchez A.H., Luthringer B.J.C., Feyerabend F., Willumeit R. (2015). Mg and Mg alloys: How comparable are in vitro and in vivo corrosion rates? A review. Acta Biomater..

[60] Körner C., Bauereiß A., Attar E. (2013). Fundamental consolidation mechanisms during selective beam melting of powders. Model. Simul. Mater. Sci. Eng..

[61] Zhao J., Gao L.-L., Gao H., Yuan X., Chen X. (2015). Biodegradable behaviour and fatigue life of ZEK100 magnesium alloy in simulated physiological environment. Fatigue Fract. Eng. Mater. Struct..

[62] Gu X.N., Zhou W.R., Zheng Y.F., Cheng Y., Wei S.C., Zhong S.P., Xi T.F., Chen L.J. (2010). Corrosion fatigue behaviors of two biomedical Mg alloys—AZ91D and WE43—In simulated body fluid. Acta Biomater..

[63] Lévesque J., Hermawan H., Dubé D., Mantovani D. (2008). Design of a pseudo-physiological test bench specific to the development of biodegradable metallic biomaterials. Acta Biomater..

